# Bacterial Vaginosis: A Comprehensive Narrative on the Etiology, Clinical Features, and Management Approach

**DOI:** 10.7759/cureus.31314

**Published:** 2022-11-10

**Authors:** Rutuja Khedkar, Sandhya Pajai

**Affiliations:** 1 Obstetrics and Gynaecology, Jawaharlal Nehru Medical College, Datta Meghe Institute of Medical Sciences, Wardha, IND

**Keywords:** hay/ison’s criteria, gardnerella, lactobacillus, probiotics, microbiome, nugent’s criteria, amsel’s criteria, bacterial vaginosis

## Abstract

Due to the intricate balance of the vaginal microbiota’s ecology, bacterial vaginosis is documented in one-third of females globally at various times of their lives. It is a typical reason for unusual vaginal discharge and is linked to various health problems. *Gardnerella vaginalis* is one of the anaerobic microorganisms linked to bacterial vaginosis. bacterial vaginosis is diagnosed by Amsel’s criteria as well as comparisons among Amsel’s criteria, Nugent’s criteria, and Hay/Ison’s criteria. To scan and assess the degree of dysbiosis within the vaginal microbiome, researchers have upped their game by combining cutting-edge molecular methods, with a focus on how specific microbial populations fluctuate in comparison to a healthy condition. A clue cell can be detected on a simple wet mount of vaginal secretions. Despite receiving regular antibiotic therapy, a substantial risk of treatment failure and bacterial vaginosis recurrence persists. Researchers have revealed positive treatment effects and reduced the infection of the female reproductive system with harmful bacteria.

## Introduction and background

There are numerous gynecological infections affecting millions of reproductive-age women globally, with bacterial vaginosis being the most common. Over the past 60 years, great research has been conducted on this medical illness, yet its pathophysiology remains unknown. The initially identified illness was characterized by a variety of mucosal inflammatory symptoms, such as secretion of vaginal fluid, pruritis, and burning sensation, as well as by an absence of leukocytic exudate, redness in the perineal region, and edema [1]. Recent research employing genetic and sophisticated culture techniques implies that what might be regarded as typical or pathological conditions happens on a diverse biological spectrum, indicating a wider microbiological variety for bacterial vaginosis than already assumed [2]. In the spectrum of putative pathogens linked to symptomatic illness, recently reported species like bacterial vaginosis-associated bacterium 1 (BVAB1), bacterial vaginosis-associated bacterium 2 (BVAB2), and bacterial vaginosis-associated bacterium 3 (BVAB3) have also been included [3]. In one assessment of publications on the chances of bacterial vaginosis, based exclusively on medical criteria, the prevalence of bacterial vaginosis ranged from 4%, as reported among non-symptomatic college-going females, to 61% of females when attended a sexually transmitted disease clinic [4]. The overabundance of normal vaginal flora is the root cause of bacterial vaginosis. Most frequently, this manifests clinically as increased, fishy-smelling vaginal discharge. The discharge itself is often of little thickness, gray, or milky in color [5]. Females who have been identified with bacterial vaginosis are at a greater risk of contracting additional sexually transmitted infections, and pregnant women are at an increased risk of giving preterm birth [6]. Bacterial vaginosis is also associated with the risk for adverse pregnancy which includes miscarriages during the second trimester, spontaneous early birth, and post-cesarean section endometritis, highlighting the importance of various review literature regarding the condition.

Bacterial vaginosis is identified as a clinical condition that occurs due to bacterial overgrowth in the vagina that leads to vaginal discharge and is characterized by a foul fishy smell with the patient most commonly complaining of itching in the perineal region. Historical facts state that *Gardnerella *vaginitis was the term used for bacterial vaginosis in the past because it was thought to be the culprit [7]. This illness is typically brought on by a reduction in the usual lactobacilli growth that produces hydrogen peroxide (H_2_O_2_) and an excess of anaerobic bacteria [8]. It was well understood that *Gardnerella vaginalis* infection usually occurs either during sexual transmission by the spread of pathogens over mucous membranes or by sharing of sexual devices. However, there is limited literature on the role of transmissibility in the case of bacterial vaginosis [9]. Some studies suggest that during sexual intercourse, the occurrence of bacterial vaginosis pathogen is due to the generated imbalance in the typical bacterial flora in the vulval region [10]. There are certain potential causes of bacterial vaginosis such as vaginal infections, multiple sexual partners, recent antibiotic use, smoking, and contraceptive use [11]. Patients usually present with complaints of foul-smelling vaginal discharge [12], which is accompanied by other clinical features, including pain or burning sensation during micturition, dyspareunia, perineal itching, redness, edema, etc., whereas in some cases, females are asymptomatic and are diagnosed during clinical examination [13] (Table 1).

**Table 1:** Comparison between a healthy vagina and bacterial vaginosis.

Healthy vaginal flora	Bacterial vaginosis
pH <4.5	pH >4.5
Presence of mature squamous epithelial cells	Presence of mature squamous epithelial cells
No amine odor present	Strong amine odor present
Absence of clue cells	Presence of clue cells
Absence of neutrophils	Absence of neutrophils
Monomorphic flora present	Polymorphic flora present
Microbiome dominated by lactobacilli seen	Microbiome dominated by *Gardnerella vaginalis*, Autopodium, and Vaginae seen

## Review

Clinical features

An odor that is typically referred to as “fishy” is the primary sign of bacterial vaginosis. The anaerobic bacteria that are responsible for this produce amines such as trimethylamine, putrescine, and cadaverine, which are responsible for this fishy smell [14]. Although that is less specific, increased vaginal discharge is a more common sign of bacterial vaginosis. Studies on symptomatic patients discovered these signs in 73% and 92% of patients, respectively [15]. If symptoms alone had been used to confirm diagnosis and therapy, 45% of research participants who had irritating symptoms (itching, burning, and pain) may have been mistaken for vaginitis caused by other conditions [15]. However, a study by Klebanoff and colleagues emphasized how unreliable symptoms are for diagnosing conditions [16]. They discovered that 57% of individuals without bacterial vaginosis and 58% of patients with bacterial vaginosis had complained of odor and discharge six months before. Hence, to confirm the diagnosis, all individuals exhibiting vaginal symptoms should be evaluated. More than 50% of those with bacterial vaginosis are asymptomatic, according to studies that employed standard screening to detect patients [14]. For diagnosing the condition by examination, the most common instrument used is per speculum examination [12].

Investigations

Sample collection is done using a sterile swab, and samples can be taken from the posterior fornix and lateral vaginal walls [12]. The swab is delivered by (cultures or dry slides) and can be transported either at room temperature or in a 40°C environment. There are certain techniques available for diagnosis; clinical criteria and lab-based testing are the two primary types of bacterial vaginosis diagnostic techniques.

Amsel’s criteria are the clinical standards that are most commonly used [17]. Three of the following four criteria must be satisfied to make this clinical diagnosis: pH of the vagina is more than 4.5; the presence of clue cells in the vulval fluid; the presence of a whitish milky uniform vulval secretion, and production of an amine fish-like odor upon adding of 10% potassium hydroxide (KOH) to the vulval discharge [17]. To rule out the more dangerous illnesses still on the differential diagnosis, it is crucial to evaluate for fever, pelvic discomfort, and a history of sexually transmitted infections [13,18] (Tables 2, 3).

**Table 2:** Diagnostic criteria for bacterial vaginosis.

Criteria	
Amsel’s diagnostic characteristics (at least three out of four to be present)	Uniform vulval fluid
	Vulval pH more than 4.5 (on litmus paper test)
	Positive Whiff test
	Clue cells seen (more than 20% of cells)
Gram-stained vulval smear (Hay/Ison) – Presence of more *Molucus *or *Gardnerella *morphotypes with little or no lactobacilli	

**Table 3:** Comparison of Amsel, Nugent, and Hay/Ison criteria (adapted from Hainer and Gibson) for bacterial vaginosis.

	Amsel’s criteria	Nugent’s criteria	Hay/Ison’s criteria
Type	Both clinical and lab investigation	Lab investigation	Lab investigation
Diagnosis time	Rapid	Long	Long
Expertise required	Clinicians	Seasoned pathologists and lab technicians	Knowledgeable laboratory technicians
Lab requirements	Low	High	Moderate
Grading system	When three of the four criteria are met, the diagnosis is considered verified: (a) Narrow, uniform grayish-white discharge is seen. (b) Vulval pH is more than 4.5 (c) Positive Whiff amine test and potassium hydroxide (KOH) test. (d) 20% or more of clue cells seen on a saline wet mount	Score 0-4: Common vegetation. Score 4-6: intermediate. Score 7 and above: Bacterial vaginosis	Category 1: Normal flora (*Lactobacillus* only) Category 2: intermediate (*Lactobacillus *= *Gardnerella*) Category 3: Bacterial vaginosis (*Lactobacillus *< *Gardnerella*)

Management

Even though up to 30% of cases of bacterial vaginosis are self-limiting, this illness can also be managed with the use of antibiotics [19]. Both of these drugs work well whether ingested or administered vaginally. Both substances can be used safely by pregnant women [19]. About 10-15% of females may need extra therapy if their condition does not improve after taking the first round of antibiotics. Partners do not need to be treated for this condition because it is not regarded as a sexually transmitted infection, and there is no chance of partner transmission [20]. It has been demonstrated that up to 80% of women who get therapy may experience a recurrence [19]. The second round of antibiotics is often administered if a patient exhibits recurring symptoms. Probiotics may be used to cure or prevent bacterial vaginosis, according to a 2009 Cochrane study, which revealed preliminary but inadequate evidence to do so [19]. According to previous studies, pregnant women with symptoms of bacterial vaginosis should get clindamycin treatment before 22 weeks of pregnancy to lower the chance of labor occurring before 37 weeks [21]. The question is whether to screen or treat bacterial vaginosis in the normal population to decrease the chances of adverse consequences such as premature birth has not been settled with great certainty. Asymptomatic women may not currently be screened for bacterial vaginosis, but symptomatic women should be tested and treated instead [21]. Both oral and vaginal versions of clindamycin are thought to be safe for use in expecting mothers [22]. Therapy among male partners was not effective in avoiding recurrent bacterial vaginosis, according to the systematic study by Mehta [23]. Orally, metronidazole (200 mg) is taken thrice a day for seven days, whereas intravaginally, there is the application of metronidazole gel (0.75% of 5 g) once a day for five days for complete treatment. Clindamycin is usually taken intravaginally as a gel (2% of 5 g) once a day for five days. The obstetric problems are shown to be prevented by vaginal application once daily for five days. Sexual partners should also be attended concurrently. A study showed that 80% of patients are cured with these medications [24].

Importance of Probiotics

Probiotics are defined as non-living substances which promote microbiota growth and provide health benefits. They appear to be an appealing probiotic alternative or co-factor (symbiotic) with probiotics in the treatment of bacterial vaginosis [25,26]. A clinical experiment in Turkey conducted in 1998 assessed the effectiveness of a symbiotic that included the probiotic *L. acidophilus* bacterial vaginosis together with estriol and lactose [26]. Women who used a prebiotic gel with APP-14 for 16 days showed improved recovery toward a normal vaginal flora, according to a 2012 study by Coste et al. containing gluco-oligosaccharides that encourage selective growth of several beneficial *Lactobacillus *species [27]. When taken orally by a healthy female, the probiotic combination Respecta®, comprising Lactoferrin RCXTM, *L. rhamnosus* HN001 (L1), and *L. acidophilus* La-14 (L2), successfully enhanced their (*Lactobacillus*) abundance in the vagina [28]. Even though this study cannot be used to draw any conclusions about therapeutic potential, Jang et al. modified the procedure used in a bacterial vaginosis animal study and discovered that Respecta®, when administered orally or intravaginally, attenuated *Gardnerella vaginalis*-induced bacterial vaginosis by reducing epithelial cell disruption and myeloperoxidase activity [28]. The effectiveness of Respecta® administered as an adjuvant with oral metronidazole therapy was subsequently also examined by Russo et al. in Romania who enrolled 48 adult females [29].

Recurrence

Studies with a prolonged follow-up have shown significant rates of recurrence even though short-term cure rates are typically similar to presently advised therapies for bacterial vaginosis [30]. After the initial episode of bacterial vaginosis has been appropriately treated with metronidazole or clindamycin, metronidazole, 0.7 % gel twice weekly for four to six months, is the recommended therapy for recurrent bacterial vaginosis. It has been demonstrated that using this technique can lead to 50% lower bacterial vaginosis recurrence [31]. Probiotics have been studied as a potential adjuvant therapy for acute bacterial vaginosis infection and as a preventive measure. Although some studies have found benefits from using probiotics, there is currently no evidence to support their use in the treatment or prevention of this ailment [32]. The outcomes of a study investigating the use of presumptive bacterial vaginosis therapy at monthly intervals to decrease bacterial vaginosis recurrence have shown some promise [33]. Previous studies have shown a link between low blood vitamin D levels and an increase in the incidence of bacterial vaginosis. Unfortunately, further research has not revealed a reduction in bacterial vaginosis recurrence with high-dose vitamin D administration [34].

Differential diagnosis

A thorough clinical examination can assist in restricting the differential diagnosis and ruling out different illnesses with similar symptoms, such as the herpes simplex virus. The cervix and vagina are inspected using a speculum to detect candidiasis or trichomoniasis which can be confirmed with a wet mount of the vulval discharge. Chlamydia and gonorrhea can be cultivated with additional cervical swabs [13]. To rule out the more dangerous illnesses still on the differential diagnosis, it is crucial to evaluate for increasing body temperature, pelvic discomfort, and previous history of sexually transmitted infections [13].

Complications

Repetitive infections, such as pelvic inflammatory diseases [24], increased the likelihood of contracting gonorrhea or chlamydia by 1.9 and 1.8 times, respectively [13]. According to researchers (research data), bacterial vaginosis with HIV-positive infected women is more likely than bacterial vaginosis with HIV-negative women to transfer HIV to their sexual partners [13]. Preterm birth during pregnancy has been linked to bacterial vaginosis, as well as a three-to-five-fold greater chance of spontaneous abortion in women diagnosed with bacterial vaginosis in the first trimester, and a two-to-three-fold higher risk of early childbirth, especially if bacterial vaginosis is discovered in the early second trimester [35].

## Conclusions

Bacterial vaginosis is mainly caused by *Gardnerella vaginalis*, and due to this, vaginal fluid is produced which has a pH of more than 4.7 and has a fishy odor when mixed with KOH solution (whiff test). The clue cells are diagnostic of bacterial vaginosis. Bacterial vaginosis is more common in symptomatic pregnant women and is linked to a history of sexually transmitted diseases, vaginal discharge, having had several sexual partners, and spontaneous miscarriage. According to Nugent’s scoring method and Amsel’s criteria, the chances of bacterial vaginosis among pregnant women are often greater than that reported in earlier research. Due to the chances of bacterial vaginosis in pregnant females, it is important to identify females who exhibit the mentioned risk factors as soon as possible. This will help avoid difficulties and ensure a successful pregnancy.
